# Sentinel Lymph Node Biopsy Predicts Non-Sentinel Lymph Node Metastases and Supports Omission of Axillary Lymph Node Dissection in Breast Cancer Patients

**DOI:** 10.3390/jcm14103441

**Published:** 2025-05-14

**Authors:** Toshihiko Yoneto, Fumiko Ikiuo, Naoko Koyanagi, Takayuki Yoshimoto, Yasutaka Takeda

**Affiliations:** 1 Department of Immunoregulation, Institute of Medical Science, Tokyo Medical University, 6-1-1 Shinjuku, Shinjuku-ku 160-8402, Tokyo, Japan; ponkichicat@mac.com; 2 Non-Invasive Clinical Cancer Therapy Research Institute, 1-8-6 Kuramae, Taito City 111-0051, Tokyo, Japan; 3 Breast Oncology Center, Double-Barred Cross Hospital, 3-1-24 Matsuyama, Kiyose 204-8522, Tokyo, Japantakeday@fukujuji.org (Y.T.)

**Keywords:** axillary lymph node dissection, breast cancer, non-sentinel lymph node, sentinel lymph node, sentinel lymph node biopsy

## Abstract

**Background:** Current international guidelines recommend omitting axillary lymph node dissection (ALND) based on sentinel lymph node biopsy (SLNB) in early-stage breast cancer patients. However, the evolving landscape of axillary management highlights the need to balance diagnostic accuracy with minimizing invasiveness. The possibility of omitting SLNB itself should also be considered. **Methods**: In this study, we have evaluated the feasibility of omitting SLNB in a total of 1044 clinically node-negative (cN (−)) breast cancer patients whose SLN status was determined by histopathology and one-step nucleic acid amplification (OSNA) after SLNB. We also analyzed SLN-positive cases to explore the association between non-SLN (NSLN) metastatic status and various biomarkers. We predicted the metastatic status of NSLNs based on patient data using a nomogram and further assessed the prevalence of macro- and micro-metastatic SLN, along with the NSLN status in SLNB cases. **Results**: Of the 644 cN (−) cases, approximately 70% of SLN-positive cases were NSLN negative, suggesting that ALND could be omitted. SLN (+) was detected approximately 7% more often by OSNA than by histopathology, suggesting that OSNA detection may be an overdiagnosis. Although NSLN-positive cases represented only 5.9% of the 581 cN (−) cases and, therefore, ALND could be omitted, it may be difficult to omit the SLNB itself as the SLN macro-metastasis was 12.5%. Biomarker analysis showed a significant correlation between total tumor load and metastatic SLN copy number with NSLN metastatic status. Based on these tumor characteristics, the nomogram predicted NSLN-positive rates very well. **Conclusions**: Thus, omitting SLNB itself carries the risk of missing high-frequency macro-metastatic SLN-positive cases and losing important SLN-related information that can predict NSLN metastases. Therefore, SLNB, which provides not only SLN status but also NSLN metastases, is necessary for reassurance in omitting ALND.

## 1. Introduction

The current standard of care for malignant disease emphasizes minimally invasive procedures, a trend that has also extended to the management of early-stage breast cancer [1]. Traditionally, axillary lymph node dissection (ALND) has played a pivotal role in achieving axillary control and predicting prognosis in breast cancer patients [2]. However, in recent years, there has been a shift toward the de-escalation of axillary surgery. Sentinel lymph node biopsy (SLNB) has become the standard approach, with studies such as the International Breast Cancer Study Group Study 23-01 (IBCSG 23-01) and the American College of Surgeons Oncology Group Z0011 (ACOSOG Z0011) showing no significant differences in recurrence or overall survival when ALND is omitted based on SLNB findings [3,4,5,6]. More recently, the SENOMAC (omission of axillary lymph node dissection in SENtinel NOde MICrometases) clinical trial demonstrated the complete safety of omitting ALND in clinically node-negative (cN (−)) breast cancer patients with sentinel lymph node (SLN) macro-metastases who received postoperative adjuvant therapy [7]. Nevertheless, concerns remain regarding the potential for overtreatment, as a high proportion of non-SLN (NSLN)-negative cases have been observed in patients undergoing ALND based on SLNB results in cN (−) cases [8,9,10]. There is ongoing debate as to whether SLNB itself, as the least invasive form of axillary surgery, can be omitted altogether [11,12,13]. However, the safety of omitting SLNB in all cN (−) cases remains to be established. The number of pathologically positive lymph nodes (pN (+)) remains a critical prognostic factor in breast cancer management, emphasizing the importance of accurate diagnosis and appropriate axillary management.

In this study, we evaluated the safety of omitting ALND, a procedure commonly performed in cN (−) breast cancer patients diagnosed as SLN positive by SLNB. In addition, we evaluated the feasibility of omitting SLNB altogether. We also analyzed SLN-positive cases to investigate the correlation between the metastatic status of NSLNs and various biomarkers. We predicted the metastatic status of NSLNs based on patient data using a nomogram and further evaluated the prevalence of macro- and micro-metastatic SLN and the NSLN status in SLNB cases. The present results suggest that it is premature to advocate omitting SLNB in all patients with cN (−) early-stage breast cancer. Rather, the SLN status determined by SLNB can predict NSLN metastases, providing more reassurance to omit ALND.

## 2. Materials and Methods

### 2.1. Patient Characteristics

This retrospective analysis included the clinicopathological data of a total of 1044 cN (−) breast cancer patients whose SLN status was assessed by both histopathology and one-step nucleic acid amplification (OSNA) between July 2006 and December 2019 (Table 1). The transition from histopathological methods to OSNA took place in April 2012 in our hospital. Patients who had received neoadjuvant chemotherapy (NAC) or had metastatic breast cancer were excluded from the study. All patients underwent axillary node assessment by ultrasound imaging, computed tomography (CT) scan, and 18F-fluorodeoxyglucose positron emission tomography/CT (18FDG-PET/CT) scan to confirm their cN (−) status. The indication criteria for SLNB were comprehensively determined by these results, and particular emphasis was placed on SUVmax ≥ 2 by PET/CT. Regarding the criteria for ALND, all SLN-positive cases were dissected until 2014, as the pathological method did not distinguish between micro-metastases and macro-metastases. After the OSNA method made it possible to distinguish between them, only macro-metastases were dissected. All cases in the study were performed by an identical team of one or two breast surgeons, diagnostic imaging physicians, and pathologists using SLNB and OSNA techniques at a single institution throughout the study period. The method used to select cases for inclusion in the study was also identical and remained unchanged throughout the study. This study was conducted in accordance with the Declaration of Helsinki and approved by the Institutional Review Board of Double-Barred Cross Hospital on 29 July 2024 with approval number 24012.

### 2.2. Histopathological Assessment

The metastatic status of SLNs was determined by histopathological methods. Excised SLNs were fixed in formalin, embedded in paraffin, sectioned at 2 mm intervals, stained with hematoxylin and eosin, and examined microscopically by a pathologist. Metastases were identified in each section. Isolated tumor cells (ITCs) were defined as instances of cancer cell infiltration into lymph nodes within 0.2 mm and were not considered metastases in this study.

### 2.3. Assessment by OSNA

The metastatic status of excised SLNs was also determined by the OSNA technique using the RD-100i system (Sysmex, Kobe, Japan) [14]. OSNA determined the metastatic status based on CK19 mRNA copy number, with results categorized as negative (<2.5 × 10^2^ copies/µL), 1 + (micro-metastasis: 2.5 × 10^2^/µL to 5.0 × 10^3^ copies/µL), or 2 + (macro-metastasis: ≥5.0 × 10^3^ copies/µL). Total tumor load was defined as the total of CK19 mRNA copy numbers of each positive SLN [15]. Although it is difficult to distinguish between macro- and micro-metastases using pathological methods, the OSNA method can clearly distinguish between them with a cut-off value of 5.0 × 10^3^ copies/µL. Since ITCs are often detected as micro-metastases, the OSNA method can discriminate the diagnosis of ITCs with a cut-off value of 2.5 × 10^2^ copies/µL. No treatment was given for ITC.

### 2.4. Prediction by Shimazu’s Nomogram

NSLN metastasis was predicted by Shimazu’s nomogram using total tumor load obtained by OSNA and tumor size as described previously [16]. Ninety-eight cases undergoing ALND of 117 SLN-positive cases from April 2012 to December 2019 were examined using Shimazu’s nomogram of NSLN-positive rate with actual rate, excluding 19 cases in which ALND was not performed due to micro-metastases.

### 2.5. Statistical Analysis

Statistical analysis was performed with the SPSS software (version 29, SPSS Inc., Chicago, IL, USA). The Mann–Whitney U test was used to compare differences between two independent groups when the dependent variable was either ordinal or continuous, but not normally distributed. The threshold for statistical significance was set at *p* < 0.05, ensuring rigorous interpretation of the results and identification of key variables influencing the metastatic status of sentinel lymph nodes.

## 3. Results

### 3.1. A Considerable Number of SLN-Positive Cases May Not Require ALND and OSNA Detection May Be an Overdiagnosis

**Figure 1:**
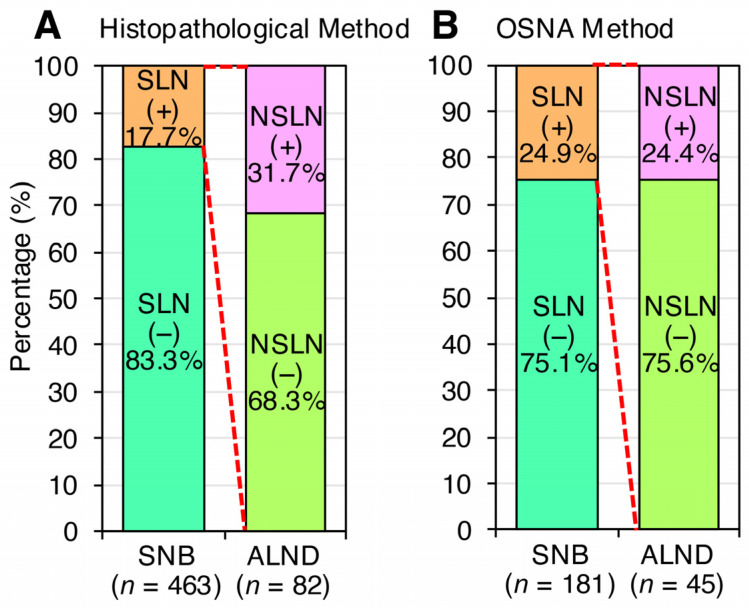
A considerable number of SLN-positive cases may not require ALND and OSNA detection may be an overdiagnosis. (**A**) Between July 2006 and March 2012, 463 cN (−) breast cancer patients who underwent mastectomy or breast-conserving surgery were assessed for SLN status by histopathological examination (Table 1). (**B**) An additional 181 cN (−) patients who underwent mastectomy or breast-conserving surgery between April 2012 and December 2014 were assessed for SLN status using OSNA (Table 1). Red dotted lines indicate that the population is the same between two columns.

Between July 2006 and March 2012, 463 cN (−) breast cancer patients who underwent mastectomy or breast-conserving surgery were assessed for SLN status by histopathological examination (Table 1). Of these, 82 (17.7%) patients were identified as SLN positive and subsequently underwent ALND. Within this group, approximately 56 (68.3%) patients were found to have negative NSLN status (Figure 1A). An additional 181 cN (−) patients who underwent mastectomy or breast-conserving surgery between April 2012 and December 2014 were assessed for SLN status using OSNA (Table 1). Of these, 45 patients (24.9%) were identified as SLN positive and underwent ALND, with approximately 34 (75.6%) patients having negative NSLN status (Figure 1B). This finding indicates that approximately 70% of SLN-positive cases that were NSLN negative underwent ALND, suggesting that a considerable number of SLN-positive cases may not require ALND.

Among all cN (−) cases, the SLN-positive rates varied, with 17.7% by histopathological examination and 24.9% by OSNA evaluation, reflecting a discrepancy of approximately 7% (Figure 1A,B). Thus, SLN (+) cases who underwent ALND were approximately 7% more often detected by OSNA than by histopathology, suggesting that OSNA detection may be an overdiagnosis. Therefore, additional information is required to increase confidence in omitting ALND. Although there are certainly some differences in background (Table 1), the subjects in this study are all cN (−), and it is important to compare pathological SLNB and SLNB by OSNA under the same criteria, partly because of the gradual spread of OSNA over time.

### 3.2. Importance of SLNB in Axillary Management

**Figure 2:**
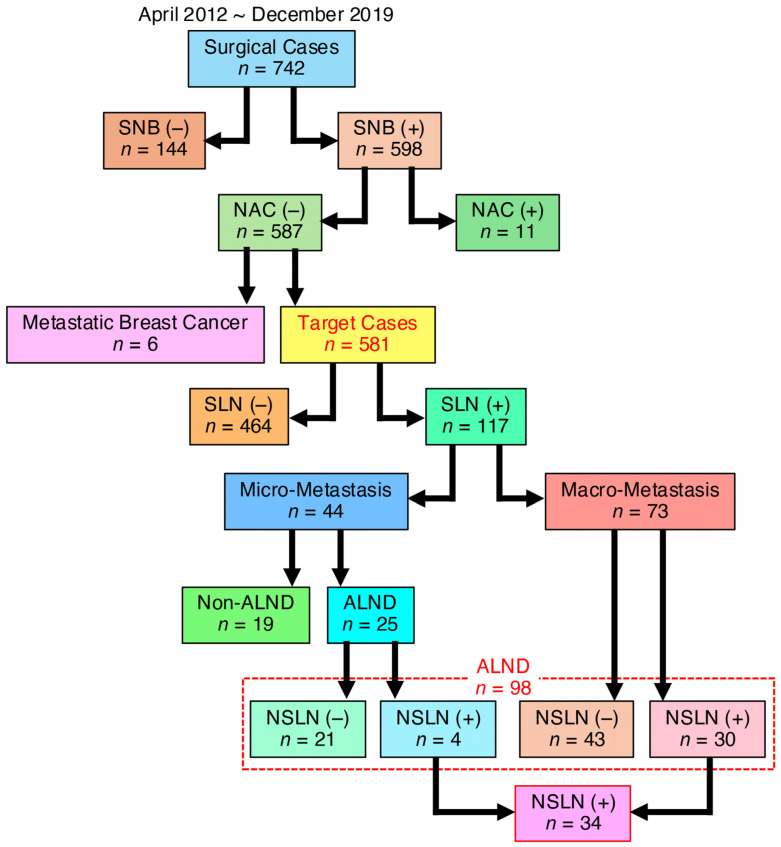
Flow of patients through treatments and number of patients studied and their distribution. Among 742 breast cancer patients evaluated between April 2012 and December 2019, 117 (20.1%) were SLN positive in a subset of 581 cN (−) cases evaluated by SLNB, excluding those who had received NAC or had metastatic breast cancer.

**Figure 3:**
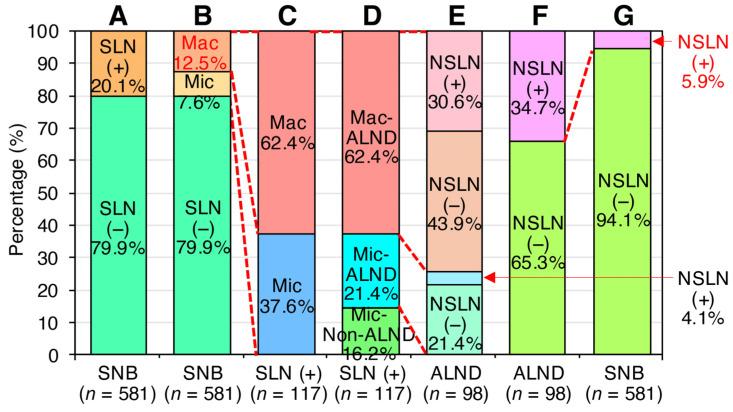
Importance of SLNB in axillary management. The SLN status in 581 cN (−) cases who underwent SLNB with OSNA from April 2012 to December 2019 (**A**) and the distribution of macro- and micro-metastases in 117 SLN-positive cases (**B**) are shown. Of 581 cN (−) cases, 117 (20.1%) were SLN positive (**A**), with 44 cases (37.6%) classified as micro-metastatic SLN positive and 73 cases (62.4%) as macro-metastatic SLN positive (**B**,**C**). Of the 117 SLN-positive cases, 98 cases underwent ALND (**D**,**E**), and 34 cases (34.7%) were found to be NSLN positive (**F**). NSLN-positive cases represented only 5.9% of the total 581 cN (−) cases (**G**). Red dotted lines indicate that the population is the same between two columns.

We next analyzed the prevalence of macro-metastatic SLN positivity in patients undergoing SLNB, as well as the rate of NSLN positivity, to assess the feasibility of omitting SLNB in all cN (−) breast cancer patients. Among 742 breast cancer patients evaluated between April 2012 and December 2019, 117 (20.1%) were SLN positive in a subset of 581 cN (−) cases evaluated by SLNB, excluding those who had received NAC or had metastatic breast cancer (Figure 2 and Figure 3A–C).

Analysis of the 117 SLN-positive cases identified by SLNB revealed that 98 cases (83.8%) proceeded to ALND based on the diagnosis of micro- and macro-metastatic SLN-positive status (Figure 3D,E). Of these, 34 cases (34.7%) were found to be NSLN positive. Notably, these 34 NSLN-positive cases represented only 5.9% of the 581 cases in which SLNB was performed (Figure 3F,G), meaning that over 90% of all cases can be locally controlled by performing SLNB alone, and, therefore, ALND could be omitted.

Moreover, of these SLN-positive cases, 44 (37.6%) were micro-metastatic SLN positive, and 73 (62.4%) were macro-metastatic SLN positive (Figure 3C). Notably, these 73 macro-metastatic SLN-positive cases represented 12.5% of the total 581 cN (−) cases (Figure 3B). Thus, if SLNBs were not performed in cN (−) cases, approximately 20% of pN (+) and 12.5% of macro-metastases would be preserved, and, therefore, it may be difficult to omit the SLNB itself.

### 3.3. Correlation of NSLN Metastasis Status with Tumor Characteristics

We further investigated the potential for omitting ALND in a subset of patients initially diagnosed as SLN positive by OSNA. The analysis focused on identifying cases within this group where ALND may be unnecessary. To accomplish this, we performed a comprehensive correlation analysis between NSLN metastatic status and various biomarkers. We investigated the relationship between NSLN metastatic status and biomarkers, such as estrogen receptor (ER), progesterone receptor (PgR), human epidermal growth factor receptor type 2 (HER2), vascular invasion, lymphatic invasion, nuclear grade, ki-67, tumor size (T-factor), histological type, patient age, the maximum copy number of metastatic SLNs, total tumor load and SLN metastasis, as determined by OSNA.

Among the 45 SLN-positive cases analyzed between April 2012 and December 2014, no significant correlations were found between NSLN metastasis status and individual factors. However, when focusing on 21 cases with micro-metastatic SLN-positive status, a significant correlation between tumor size and NSLN metastasis status was observed (Table 2). This finding suggests that it may be possible to omit ALND in cN (−) cases with micro-SLN-positive status and a primary tumor diameter of less than 2 cm. Originally, 181 SLNB cases were included in this study, partly because micro-metastases were rare in SLNB cases. The reason why the number of cases has not increased from 21 since then is that it has become standard practice not to dissect cases with micro-metastases. However, the study of these 21 cases suggests that the presence or absence of NSLN metastases is related to the tumor volume of the cancer cells rather than to the nature of the cancer cells, such as biomarkers, and this has implications for the subsequent Shimazu’s nomogram.

Furthermore, after expanding the cohort to 69 SLN-positive cases from April 2012 to December 2016, including macro-metastatic SLN-positive cases, total tumor load, maximum copy number of metastatic SLN, and SLN metastasis emerged as significant factors associated with NSLN metastasis status (Table 3). This analysis highlights the importance of these factors in predicting NSLN metastatic status within this cohort. These results suggest that tumor size and the degree of SLN metastasis (referred to as “tumor pressure”) may have a greater influence on NSLN metastasis than the biological characteristics of the tumor, such as biomarker status.

### 3.4. Prediction of NSLN Metastatic Status from Tumor Characteristics Using Shimazu’s Nomograms

**Figure 4:**
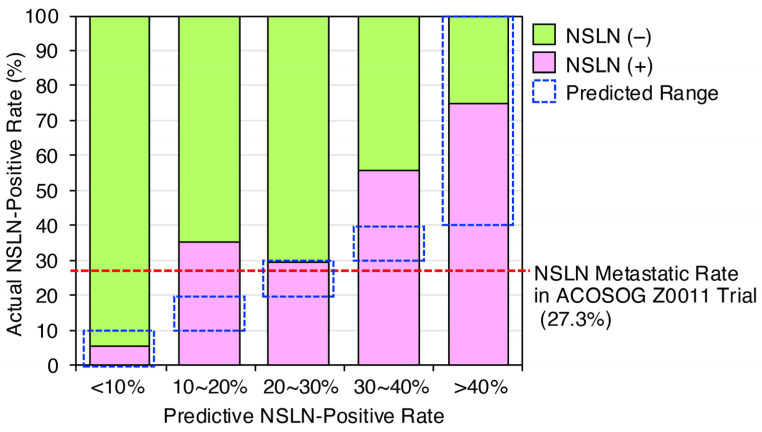
Prediction of NSLN metastasis status by SLN information using the Shimazu’s nomogram. After the introduction of OSNA, 98 of 117 SLN-positive cases from April 2012 to December 2019 were evaluated by Shimazu’s nomogram, which used the total tumor load and primary tumor diameter, to compare the NSLN-positive rate with the actual rate, excluding 19 cases in which ALND was not performed due to micro-metastases. A dotted square indicates the predicted range. In the ALND group of the ACOSOG Z0011 clinical trial, 27.3% of patients were reported to have additional metastases in non-SLNs removed by ALND [6].

To assess the predictive ability of NSLN metastases from SLN status, we finally used Shimazu’s nomograms, which were calculated based on preoperative and intraoperative factors [16]. We predicted NSLN metastatic status based on tumor characteristics, such as total tumor load and primary tumor diameter, in 98 cases, excluding 19 non-ALND cases with micro-metastatic SLN-positive status out of 117 SLN-positive cases in 581 cN (−) cases from April 2012 to December 2019 (Figure 2). The 19 cases were excluded because it has become standard practice to omit dissection for micro-metastases. Notably, the predicted values correlated well with the actual results (Figure 4), supporting the importance of SLN information to predict NSLN metastasis status. Thus, the metastatic status of NSLNs can be predicted from the SLN information, such as tumor size and extent of SLN metastasis obtained by SLNB, further demonstrating the importance of SLNB in axillary management.

## 4. Discussion

In recent years, breast cancer treatment has increasingly focused on minimizing invasiveness while optimizing patient outcomes. A key component of this shift is the de-escalation of ALND and the adoption of SLNB as a critical diagnostic tool in cN (−) breast cancer cases [17,18]. The historical importance of ALND in breast cancer staging and prognosis is well documented. However, several clinical trials have shown that omitting ALND based on SLNB results does not compromise overall survival or increase recurrence rates [4,5,19,20,21]. This paradigm shift is consistent with the broader trend toward less invasive approaches to breast cancer treatment, including the potential omission of SLNB itself [11,12,13].

In this study, we reassessed the validity of omitting ALND based on SLNB findings in cN (−) patients. Current international consensus guidelines recommend omitting ALND in cN (−) cases with SLN-positive findings detected by SLNB. Our analysis showed that if cN (−) cases with micro-SLN-positive findings who did not undergo ALND were assumed to be NSLN-negative cases, the number of NSLN-positive cases was only 5.9% of all cN (−) cases, a very small proportion (Figure 3). Considering the axillary recurrence rate of a few percent, as reported in the National Surgical Adjuvant Breast and Bowel Project (NSABP) B-04 trial, the addition of postoperative treatments, such as radiation, reduces concerns about clinical outcomes [21]. These findings reaffirm the validity of omitting ALND in SLN-positive cases. We investigated whether differences in SLNB results obtained by pathological examination and OSNA in cN (−) patients could lead to unnecessary ALND. Our study found a higher rate of NSLN-negative cases when using OSNA-based SLNB compared to pathology-based SLNB (Figure 1). This suggests that micro-metastases may be missed by pathological examination using 2 mm interval sections, whereas OSNA-based SLNB can detect these micro-metastases, thereby increasing the number of SLN-positive cases and, consequently, the number of cases in which ALND is performed. This, in turn, increases the relative NSLN-negative rate. Thus, OSNA-based SLNB may lead to overtreatment. To mitigate this, we analyzed whether it is possible to predict NSLN metastasis in cases diagnosed as SLN positive by OSNA. Our analysis identified potential determinants of NSLN metastasis in SLN-positive cases, including tumor size, total tumor load, maximum copy number of metastatic SLNs, and SLN metastasis (Table 2 and Table 3). This suggests the potential feasibility of predicting NSLN metastasis based on SLN information (Figure 4).

Regarding the economic impact of SLNB alone compared to traditional ALND, traditional ALND is considered a burden in terms of both healthcare costs and patient quality of life, with longer operation times, longer hospital stays and the risk of postoperative complications (lymphedema, reduced mobility, etc.). With the introduction of OSNA, the detection of SLN metastases, especially micro-metastases, is more sensitive than conventional tissue diagnosis, but the high sensitivity of this method is considered overdiagnostic due to the risk of overtreatment, including an increased false-positive rate and unnecessary ALNDs. This study highlights the possibility of avoiding further unnecessary ALNDs, thereby increasing their economic benefit, if NSLN-negative results can be assessed.

While this study suggests that SLNB is critical for assessing not only SLN status but also the presence of NSLN metastases, there are several limitations that need to be acknowledged and addressed in future research. First, this is a retrospective study, and therefore, bias due to differences in post-operative treatment and other factors may be introduced into the study. Secondly, because we started to introduce OSNA in our institution in line with the worldwide introduction of OSNA, the timing of SLNB determination by the pathological diagnosis method and the OSNA method was different and had to be divided into two periods. As a result, the method of presenting the results became complicated and the homogeneity of the sample may have been affected. Thirdly, the present data were evaluated only by the univariate analysis (Mann–Whitney U test) and not by the multivariate analysis. In addition, we do not have comparative data on the long-term prognosis between OSNA and pathological methods; however, we believe that the OSNA method does not have a worse prognosis than the pathological method, as it results in more axillary dissections being indicated. Furthermore, the correlation with postoperative adjuvant therapies, such as adjuvant chemotherapy and radiotherapy, which are indeed important factors in the risk of recurrence and the safety of omitting ALND, was not addressed.

The various nomograms using the extent of lymph node metastasis and primary tumor diameter successfully predict NSLN metastatic status and serve as useful tools for clinical decision making [22,23]. Unlike other predictive tools that incorporate postoperative pathological factors, our study used Shimazu’s nomogram [16]. In SLNB-positive patients, intra-operative prediction of NSLN metastatic risk using Shimazu’s nomograms allows determination of metastatic risk and rapid intra-operative treatment decisions. Although our predicted values were slightly lower than the actual values, setting the cut-off in the nomogram at 30% and performing ALND on cases with a predicted value of 30% or higher would leave 19 (3.3%) NSLN-positive cases untreated (Figure 4). Lowering the cut-off to 10% and performing ALND on cases with a predicted value of 10% or higher would reduce the number of untreated NSLN-positive cases to just one (0.2%). This suggests that lowering the cut-off may increase the sensitivity of the nomogram, thereby reducing the number of missed NSLN-positive cases. However, it also implies that the number of patients undergoing unnecessary ALND may increase, highlighting the importance of a balanced approach when setting the cut-off. The integration of such predictive models into clinical practice could improve the safety and effectiveness of omitting ALND in cases diagnosed as SLN positive by OSNA. Thus, a low cut-off threshold increases the number of patients who can avoid ALND and reduces complications (e.g., lymphedema), but increases the risk of missing NSLN metastases. A higher threshold also results in a lower risk of recurrence, but also increases the number of patients for whom ALND is unnecessary. Thus, although some usefulness of the nomogram has been demonstrated, its practical application, including cut-off values in real-world clinical practice, is considered to be an issue for the future.

Based on these results, we further investigated the feasibility of omitting SLNB in all cN (−) breast cancer patients. Recent studies have focused on omitting axillary surgery, including SLNB, in specific subgroups of breast cancer patients. For example, the SOUND trial investigated the feasibility of omitting SLNB in postmenopausal hormone receptor positive, HER2-negative breast cancer patients eligible for breast-conserving surgery with a negative preoperative axillary ultrasound and found no adverse effect on 5-year distant disease-free survival [24,25]. Similarly, the Society of Surgical Oncology recommends against routine SLNB in cN (−), HR-positive and HER2-negative breast cancer patients aged 70 years or older [26] (https://www.choosingwisely.org/). However, a direct comparison of outcomes between patients with and without SLNB in a broader population is lacking. Our data showed that omitting SLNB in 581 cN (−) cases would result in 117 pN (+) cases (20.1%), including 73 macro-SLN-positive cases, representing 12.5% of cN (−) cases among all cN (−) cases (Figure 3). This finding implies that omitting SLNB in all cN (−) cases could result in a significant number of undetected macro-metastatic SLN-positive cases, highlighting the critical role of SLNB in axillary management. Comparing the results of the SOUND trial with our data, the proportion of NSLN-positive cases among SLN-positive cases was similar (3.9% in SOUND vs. 5.9% in our study), and the proportion of pN-positive cases was almost identical (22.7% in SOUND vs. 20.1% in our study) (Figure 3). Notably, these pN (+) rates exceed the previously accepted false-negative rate of 10% for SLNB [27] and are outside acceptable criteria.

Therefore, while omission of SLNB may be feasible in select cases, it is premature to consider omission of SLNB in all cN (−) cases at this time. Recent advances in axillary management highlight the need to balance diagnostic accuracy with minimizing patient invasiveness to achieve favorable outcomes. Clinical trials, such as the SENOMAC trial, provide evidence to support omitting ALND based on SLNB findings [7], while trials, such as the SOUND trial, validate omitting SLNB in certain patient groups [24,25]. Recent studies and guidelines have thus highlighted the option of omitting SLNB in clinically node-negative (cN (−)) patients. However, the actual cases in which it can be omitted are limited to those with a primary tumor diameter of 2 cm or less or other indications. Therefore, SLNB is considered essential at this stage of axillary assessment, as it allows a more accurate assessment of NSLNs and is particularly useful in detecting macro-metastases. Consistent with this study, a recent report of a sub-analysis of the randomized SINODAR-ONE clinical trial showed that in patients with T1–2 breast cancer and one to two macro-metastatic SLNs treated with total mastectomy, the overall survival and recurrence rates of patients treated with SLNB alone were not inferior to those treated with ALND [28]. As post-operative radiotherapy is omitted in the case of total mastectomy, the usefulness of NSLN prediction by SLNB will become clearer. However, the analysis presented here highlights the complexities and nuances involved in making decisions about axillary management. The ultimate aim of our study is not to debate the pros and cons of omitting SLNB. Rather, our focus is to assess the diagnostic accuracy of SLNB and the role of OSNA in the assessment of macro-metastases. Although the active omission of SLNB is an area of current debate, SLNB remains an important tool in clinical practice, even in cN (−) cases with a risk of macro-metastases. Our findings reinforce the importance of SLNB in providing both absolute reassurance and predictive information in the diagnosis of non-SLN metastases.

## 5. Conclusions

In conclusion, given that approximately 20% of metastatic axillary lymph nodes remain undetected in the cN (−) case group in this study, and that critical information influencing NSLN metastasis predictors cannot be obtained without SLNB, it is premature to universally omit SLNB in all patients. We propose to adopt more advanced approaches to predict NSLN metastasis based on SLNB data, focusing in particular on total tumor load and maximum copy number of metastatic SLNs detected by OSNA. In the future, improving predictive tools and imaging diagnostics for axillary lymph node metastasis will be essential for integrating these emerging technologies and refining axillary management strategies to ensure optimal outcomes for breast cancer patients.

## Figures and Tables

**Table 1:** SLNB-performed patient information and tumor characteristics. 463 cN (−) patients who underwent SLNB using the histological method between July 2006 and March 2012 and 581 cN (−) cases who underwent SLNB using the OSNA method between April 2012 and December 2019 were compared.

Characteristics		Histological Method		OSNA Method							
		SLNB (+)		SLNB (+)		SLN (+) Micro- Metastasis		SLN (+) ALND- Performed		NAC (−) Metastasis (−)	
Period		July 2006 ~ March 2012		April 2012 ~ December 2014		April 2012 ~ December 2014		April 2012 ~ December 2016		April 2012 ~ December 2019	
Total patient number		*n* = 463		*n* = 181		*n* = 21		*n* = 69		*n* = 581	
Age (Mean ± SD)		61.6 ± 12.7		62.2 ± 13.9		64.5 ± 12.3		62.0 ± 14.0		63.2 ± 13.0	
Patient number, *n*, and %		*n*	%	*n*	%	*n*	%	*n*	%	*n*	%
Gender	Male	4	0.9	0	0	0	0	0	0	1	0.2
	Female	459	99.1	181	100	21	100	69	100	580	99.8
Tumor stage	Tis	40	8.6	15	8.3	0	0	1	1.4	65	11.2
	T1	270	58.3	117	64.6	11	52.4	42	60.9	392	67.5
	T2	131	28.3	44	24.3	9	42.9	23	33.3	111	19.1
	T3	10	2.2	3	1.7	1	4.8	3	4.3	16	2.8
	T4	9	1.9	1	0.6	0	0	0	0	1	0.2
	Missing data	3	0.6	1	0.6	0	0	0	0	6	1.0
Nottingham histologic grade	Grade 1	173	37.4	83	45.9	10	47.6	43	62.3	377	64.9
	Grade 2	104	22.5	48	26.5	6	28.6	11	15.9	100	17.2
	Grade 3	145	31.3	47	26.0	5	23.8	15	21.7	95	16.4
	Missing data	41	8.9	3	1.7	0	0	0	0	9	1.5
Tumor subtype	ER (+), HER2 (−)	242	52.3	84	46.4	11	52.4	29	42.0	165	28.4
	ER (+), HER2 (+)	129	27.9	67	37.0	7	33.3	33	47.8	325	55.9
	ER (−), HER2 (+)	49	10.6	18	9.9	2	9.5	6	8.7	62	10.7
	ER (−), HER2 (−)	33	7.1	11	6.1	1	4.8	1	1.4	29	5.0
	Missing data	10	2.2	1	0.6	0	0	0	0	0	0
Type of breast surgery	Mastectomy	192	41.5	95	52.5	14	66.7	52	75.4	363	62.5
	Breast-conserving surgery	271	58.5	86	47.5	7	33.3	17	24.6	218	37.5

**Table 2:** Tumor size is a potential determinant of NSLN metastasis in SLN micro-metastasis-positive cases. Characteristics of tumors, including biomarkers (ER, PgR and HER2), nuclear grade, Ki-67, subtype, tumor size, histological type, age, lymphatic invasion, vascular invasion, and total tumor load were compared with NSLN metastatic status in 21 SLN micro-metastasis-positive cases between April 2012 and December 2014. Of 181 cN (−) cases, 45 SLN-positive cases underwent ALND, including 21 SLN micro-metastasis-positive cases. *p*-values were determined by the Mann–Whitney U test. * *p* < 0.05.

Characteristics		*n*		*p*-Value
		NSLN		
		(+)	(−)	
ER	(+)	2	16	0.546
	(−)	1	2	
PgR	(+)	1	12	0.366
	(−)	2	6	
HER2	(+)	1	3	0.651
	(−)	2	15	
Nuclear grade	1	1	9	0.291
	2	0	6	
	3	2	3	
Ki-67	≤10%	2	10	0.651
	10~30%	1	5	
	≥30%	0	3	
Subtype	Luminal A	1	12	0.615
	Luminal B	2	2	
	Luminal B/HER2	0	2	
	HER2	0	1	
	Triple negative	0	1	
Tumor size	1	0	11	0.044 *
	2	2	7	
	3	1	0	
Histological type	Tubular	0	9	0.159
	Solid	1	3	
	Scirrhous	1	4	
	Others	1	1	
Age	<50	0	2	0.315
	50~70	1	12	
	>70	2	4	
Lymphatic invasion	(+)	3	12	0.366
	(−)	0	6	
Vascular invasion	(+)	3	5	0.050
	(−)	0	13	
Total tumor load	<1000	1	11	0.580
	1000~2500	2	5	
	2500~5000	0	2	

**Table 3:** Maximum copy number of metastatic SLNs, total tumor load, and SLN metastasis are potential determinants of NSLN metastasis in SLN-positive cases. Tumor characteristics, including biomarkers (ER, PgR and HER2), vascular invasion, lymphatic invasion, nuclear grade, Ki-67, tumor size, maximum copy number of metastatic SLNs, total tumor load, and SLN metastasis were compared with NSLN metastatic status in 69 SLN-positive cases undergoing ALND, between April 2012 and December 2016. p-values were determined by the Mann–Whitney U test. * *p* < 0.05, ** *p* < 0.01.

Characteristics		*n*		*p*-Value
		NSLN		
		(+)	(−)	
ER	(+)	18	43	0.472
	(−)	3	4	
PgR	(+)	15	34	0.991
	(−)	4	9	
HER2	(+)	4	6	0.472
	(−)	16	40	
Vascular invasion	(+)	7	10	0.373
	(−)	15	36	
Lymphatic invasion	(+)	15	23	0.189
	(−)	7	22	
Nuclear grade	1	10	32	0.093
	2	4	7	
	3	7	8	
Ki-67	≤10%	7	17	0.629
	10%~30%	8	20	
	≥30%	6	10	
Tumor size	is	0	1	0.069
	1	11	31	
	2	8	15	
	3	3	0	
Maximum copy number of metastatic SLN (cps/µL)	<1 × 10^4^	6	28	0.002 **
	~2 × 10^4^	1	5	
	~4 × 10^4^	2	3	
	~10 × 10^4^	3	5	
	>10 × 10^4^	10	6	
Total tumor load	<1 × 10^4^	6	26	0.004 **
	~2 × 10^4^	1	5	
	~4 × 10^4^	2	5	
	~10 × 10^4^	3	5	
	>10 × 10^4^	10	6	
SLN metastasis	Micro	4	21	0.034 *
	Macro	18	26	

## Data Availability

The data that support the findings of this study are available from the authors upon reasonable request.

## Abbreviations

The following abbreviations are used in this manuscript:

ALND	Axillary lymph node dissection
CT	Computed tomography
cN (−)	Clinically node negative
ER	Estrogen receptor
HER2	Human epidermal growth factor receptor type 2
ITC	Isolated tumor cell
NAC	Neoadjuvant chemotherapy
NSLN	Non-sentinel lymph node
OSNA	One-step nucleic acid amplification
pN (+)	Pathologically positive lymph node
PgR	Progesterone receptor
SLN	Sentinel lymph node
SLNB	Sentinel lymph node biopsy

## References

[1] Ngai V., Tai J.C.J., Taj S., Khanfar H., Sfakianakis E., Bakalis A., Baker R., Ahmed M. (2022). Non-invasive predictors of axillary lymph node burden in breast cancer: A single-institution retrospective analysis. Breast Cancer Res. Treat..

[2] Nemoto T., Vana J., Bedwani R.N., Baker H.W., McGregor F.H., Murphy G.P. (1980). Management and survival of female breast cancer: Results of a national survey by the American College of Surgeons. Cancer.

[3] Galimberti V., Cole B.F., Zurrida S., Viale G., Luini A., Veronesi P., Baratella P., Chifu C., Sargenti M., Intra M. (2013). Axillary dissection versus no axillary dissection in patients with sentinel-node micrometastases (IBCSG 23-01): A phase 3 randomised controlled trial. Lancet Oncol..

[4] Galimberti V., Cole B.F., Viale G., Veronesi P., Vicini E., Intra M., Mazzarol G., Massarut S., Zgajnar J., Taffurelli M. (2018). Axillary dissection versus no axillary dissection in patients with breast cancer and sentinel-node micrometastases (IBCSG 23-01): 10-year follow-up of a randomised, controlled phase 3 trial. Lancet Oncol..

[5] Giuliano A.E., Ballman K.V., McCall L., Beitsch P.D., Brennan M.B., Kelemen P.R., Ollila D.W., Hansen N.M., Whitworth P.W., Blumencranz P.W. (2017). Effect of Axillary Dissection vs. No Axillary Dissection on 10-Year Overall Survival Among Women With Invasive Breast Cancer and Sentinel Node Metastasis: The ACOSOG Z0011 (Alliance) Randomized Clinical Trial. JAMA.

[6] Giuliano A.E., Hunt K.K., Ballman K.V., Beitsch P.D., Whitworth P.W., Blumencranz P.W., Leitch A.M., Saha S., McCall L.M., Morrow M. (2011). Axillary dissection vs. no axillary dissection in women with invasive breast cancer and sentinel node metastasis: A randomized clinical trial. JAMA.

[7] de Boniface J., Filtenborg Tvedskov T., Ryden L., Szulkin R., Reimer T., Kuhn T., Kontos M., Gentilini O.D., Olofsson Bagge R., Sund M. (2024). Omitting Axillary Dissection in Breast Cancer with Sentinel-Node Metastases. N. Engl. J. Med..

[8] Dingemans S.A., de Rooij P.D., van der Vuurst de Vries R.M., Budel L.M., Contant C.M., van der Pool A.E. (2016). Validation of Six Nomograms for Predicting Non-sentinel Lymph Node Metastases in a Dutch Breast Cancer Population. Ann. Surg. Oncol..

[9] Majid S., Ryden L., Manjer J. (2019). Determinants for non-sentinel node metastases in primary invasive breast cancer: A population-based cohort study of 602 consecutive patients with sentinel node metastases. BMC Cancer.

[10] Wang X., Zhang G., Zuo Z., Zhu Q., Wu S., Zhou Y., Mao F., Lin Y., Shen S., Zhang X. (2022). Sentinel Lymph Node Positive Rate Predicts Non-Sentinel Lymph Node Metastasis in Breast Cancer. J. Surg. Res..

[11] Gera R., Kasem A., Mokbel K. (2018). Can Complete Axillary Node Dissection Be Safely Omitted in Patients with Early Breast Cancer When the Sentinel Node Biopsy Is Positive for Malignancy? An Update for Clinical Practice. In Vivo.

[12] Jatoi I., Kunkler I.H. (2021). Omission of sentinel node biopsy for breast cancer: Historical context and future perspectives on a modern controversy. Cancer.

[13] Reimer T. (2023). Omission of axillary sentinel lymph node biopsy in early invasive breast cancer. Breast.

[14] Huxley N., Jones-Hughes T., Coelho H., Snowsill T., Cooper C., Meng Y., Hyde C., Mujica-Mota R. (2015). A systematic review and economic evaluation of intraoperative tests [RD-100i one-step nucleic acid amplification (OSNA) system and Metasin test] for detecting sentinel lymph node metastases in breast cancer. Health Technol. Assess..

[15] Peg V., Espinosa-Bravo M., Vieites B., Vilardell F., Antunez J.R., de Salas M.S., Delgado-Sanchez J.J., Pinto W., Gozalbo F., Petit A. (2013). Intraoperative molecular analysis of total tumor load in sentinel lymph node: A new predictor of axillary status in early breast cancer patients. Breast Cancer Res. Treat..

[16] Shimazu K., Sato N., Ogiya A., Sota Y., Yotsumoto D., Ishikawa T., Nakamura S., Kinoshita T., Tsuda H., Ohi Y. (2018). Intraoperative Nomograms, Based on One-Step Nucleic Acid Amplification, for Prediction of Non-sentinel Node Metastasis and Four or More Axillary Node Metastases in Breast Cancer Patients with Sentinel Node Metastasis. Ann. Surg. Oncol..

[17] Magnoni F., Galimberti V., Corso G., Intra M., Sacchini V., Veronesi P. (2020). Axillary surgery in breast cancer: An updated historical perspective. Semin. Oncol..

[18] Donker M., van Tienhoven G., Straver M.E., Meijnen P., van de Velde C.J., Mansel R.E., Cataliotti L., Westenberg A.H., Klinkenbijl J.H., Orzalesi L. (2014). Radiotherapy or surgery of the axilla after a positive sentinel node in breast cancer (EORTC 10981-22023 AMAROS): A randomised, multicentre, open-label, phase 3 non-inferiority trial. Lancet Oncol..

[19] Veronesi U., Paganelli G., Viale G., Luini A., Zurrida S., Galimberti V., Intra M., Veronesi P., Robertson C., Maisonneuve P. (2003). A randomized comparison of sentinel-node biopsy with routine axillary dissection in breast cancer. N. Engl. J. Med..

[20] Huang T.W., Su C.M., Tam K.W. (2021). Axillary Management in Women with Early Breast Cancer and Limited Sentinel Node Metastasis: A Systematic Review and Metaanalysis of Real-World Evidence in the Post-ACOSOG Z0011 Era. Ann. Surg. Oncol..

[21] Fisher B., Redmond C., Fisher E.R., Bauer M., Wolmark N., Wickerham D.L., Deutsch M., Montague E., Margolese R., Foster R. (1985). Ten-year results of a randomized clinical trial comparing radical mastectomy and total mastectomy with or without radiation. N. Engl. J. Med..

[22] Jimbo K., Kinoshita T., Ogura T., Watase C., Murata T., Shiino S., Takayama S., Yoshida M. (2020). Prediction score model for non-sentinel and four or more nodal metastases using a combined method of one-step nucleic acid amplification and histology in sentinel node-positive breast cancer patients. Eur. J. Surg. Oncol..

[23] Van Zee K.J., Manasseh D.M., Bevilacqua J.L., Boolbol S.K., Fey J.V., Tan L.K., Borgen P.I., Cody H.S., Kattan M.W. (2003). A nomogram for predicting the likelihood of additional nodal metastases in breast cancer patients with a positive sentinel node biopsy. Ann. Surg. Oncol..

[24] Gentilini O., Veronesi U. (2012). Abandoning sentinel lymph node biopsy in early breast cancer? A new trial in progress at the European Institute of Oncology of Milan (SOUND: Sentinel node vs Observation after axillary UltraSouND). Breast.

[25] Gentilini O.D., Botteri E., Sangalli C., Galimberti V., Porpiglia M., Agresti R., Luini A., Viale G., Cassano E., Peradze N. (2023). Sentinel Lymph Node Biopsy vs No Axillary Surgery in Patients with Small Breast Cancer and Negative Results on Ultrasonography of Axillary Lymph Nodes: The SOUND Randomized Clinical Trial. JAMA Oncol..

[26] Carleton N., Zou J., Fang Y., Koscumb S.E., Shah O.S., Chen F., Beriwal S., Diego E.J., Brufsky A.M., Oesterreich S. (2021). Outcomes After Sentinel Lymph Node Biopsy and Radiotherapy in Older Women with Early-Stage, Estrogen Receptor-Positive Breast Cancer. JAMA Netw. Open.

[27] Rozenberg S., Liebens F., Ham H. (1999). The sentinel node in breast cancer: Acceptable false-negative rate. Lancet.

[28] Gentile D., Tinterri C. (2024). Sentinel lymph node biopsy versus axillary lymph node dissection in breast cancer patients undergoing mastectomy. Minerva Surg..

